# State-of-the-Art on the Impact of Bimodal Acoustic Stimulation on Speech Perception in Noise in Adults: A Systematic Review

**DOI:** 10.3390/audiolres14050077

**Published:** 2024-10-16

**Authors:** Antonio Casarella, Anna Notaro, Carla Laria, Nicola Serra, Elisabetta Genovese, Rita Malesci, Gennaro Auletta, Anna Rita Fetoni

**Affiliations:** 1 Department of Neuroscience, Reproductive Sciences and Dentistry, Audiology Section, University of Naples Federico II, Via Pansini 5, 80131 Naples, Italy; antoniocasarella29@gmail.com (A.C.); dott.ssanotaroanna@gmail.com (A.N.); rita.malesci@unina.it (R.M.); auletta@unina.it (G.A.); annarita.fetoni@unina.it (A.R.F.); 2 Department of Diagnostic, Clinical and Public Health Medicine, Audiology Program University of Modena and Reggio Emilia, Via Del Pozzo 71, 41124 Modena, Italy; elisabetta.genovese@unimore.it

**Keywords:** bimodal stimulation, speech perception, noise, new technologies

## Abstract

Background/Objectives: Bimodal stimulation (BS), which combines the use of a cochlear implant (CI) in one ear and a hearing aid (HA) in the opposite ear, is an established strategy to treat hearing loss by exploiting the unique capabilities of each device. CIs stimulate the auditory nerve by bypassing damaged hair cells, while HAs amplify sounds by requiring a functional hearing residual. The aim of this systematic review is to investigate the advantages and disadvantages of BS such as speech perception in noise. Methods: We examined clinical studies published from October 2020 to July 2024, following the PRISMA guidelines, focusing on the advantages and disadvantages of BS on speech perception in noise in adulthood. Results: BS in adult patients significantly improves speech perception in quiet and noisy environments, especially for those with increased residual hearing. Unilateral CIs and BS perform similarly in quiet conditions, but BS significantly improves speech discrimination in noisy environments if loudness between the two devices is appropriately balanced. Conclusions: Directional microphones and programming software are new technologies that succeed in reducing environmental noise and improving verbal perception outcomes, although their features in the literature are controversial. In addition, the individuals using BS may face temporal mismatches mainly due to differing device latencies, affecting sound localization. Compensating for these mismatches can enhance localization accuracy. However, modulated noise remains a significant obstacle to verbal perception in noise. Valuable assessment tools such as music tests provide further information on hearing performance and quality of life. More research is needed to define certain selection criteria.

## 1. Introduction

Bimodal stimulation (BS), combining a cochlear implant (CI) in one ear and a hearing aid (HA) in the contralateral ear, is increasingly a strategy in the treatment of both asymmetrical and symmetrical hearing loss. This rehabilitation strategy aims to exploit the unique capabilities of each device to improve the adult patient’s overall hearing perception, particularly in noisy environments. Although the devices have different mechanisms of action, namely, the CI bypasses damaged hair cells in the inner ear and stimulates the nerve directly while HA amplifies sounds and therefore requires a functional hearing residual, they aim at the same goal in improving verbal perception. However, verbal perception in noise represents one of the most significant challenges for individuals with hearing loss, thus the BS is considered a positive solution in adults especially with residual hearing in the opposite ear [1]. It is well known that in quiet conditions, there are no significant differences between the use of a unilateral CI and the application of BS. However, in noisy conditions, this difference increases considerably in favor of BS because the ability to discriminate verbal signals is significantly increased. In addition, a recent study shows that balancing loudness between the two devices in BS leads to an improvement in speech recognition [2]. Subjects treated with BS suffer from mismatches in stimulation timing due to different processing latencies of both devices including three different dimensions: level, frequency, and latency of stimulation between the CI and HA. The latency-related delay mismatch leads to a temporal mismatch in auditory nerve stimulation. The authors showed that the device delay control mismatch affects the auditory nerve stimulation mismatch, leading to better source localization accuracy [3]. This effect is attributed to the binaural fusion of auditory information, which brings into play mechanisms for integrating signals from both ears centrally, thus improving the analysis of the speech signal regarding background noise [4,5]. When addressing the following results, it is necessary to define the concept of electric and acoustic stimulation (EAS). EAS represents the combination of two technologies: CI technology for high-pitched sounds and HA technology for low-pitched sounds. EAS aims to provide a broader range of auditory input by utilizing the strengths of both technologies, enhancing overall speech perception and hearing outcomes. A study comparing CI alone, BS (CI+HA), and best-aided EAS (CIHA+HA) users separately has demonstrated that both BS and best-aided EAS conditions showed significant benefits over CI alone, although BS (CI+HA) is the most recommended approach [6,7,8,9]. The aim of this review is to examine in detail the advantages and disadvantages of BS in adult patients on speech perception in noisy environments, to provide a comprehensive overview of the current evidence, and to suggest possible future directions for research and clinical practice.

## 2. Materials and Methods

The systematic review was conducted following the Preferred Reporting Items for Systematic Review and Meta-Analysis (PRISMA 2020). The PRISMA statement 2020 [10]. The study protocol has been stored at the following link: “https://osf.io/fbk98 (accessed on 13 August 2024)”, with the following registration doi: https://doi.org/10.17605/OSF.IO/FBK98. The research question for this systematic review was: “advantages and disadvantages of BS such as speech perception in noise”.

### 2.1. Inclusion Criteria and Eligibility

The literature review process was conducted in the following steps: identification of the research questions through the PIOS (Population, Intervention, Outcome, Design) method, literature search, included papers selection, findings appraisal, and summary building. Eligible studies should meet the following criteria: (1) population: adult population; (2) intervention: BS; (3) outcome: effectiveness in improving sensory integration or speech perception in noise; and (4) design: randomized controlled trials (RCTs) or cohort studies.

Theses, posters, commentaries, letters to the editor, reviews, and books were excluded, as well as papers whose full text was not available. Finally, we only considered English-language peer-reviewed articles.

### 2.2. Search Strategy

To identify clinical studies on verbal perception in noise in patients with BS, the manuscripts were searched on PubMed Central (National Center for Biotechnology Information, Bethesda, MD, USA), Cochrane Review (Cochrane, London, UK), and Scopus. The search strings used and the number of articles found were reported in Table 1. The literature search was conducted in July 2024.

### 2.3. Study Selection and Data Extraction

Two independent authors with more than 5 years of experience (A.C., A.N.) screened titles and abstracts according to the search strategy focused on the relationship between [(bimodal acoustic stimulation)] and [(speech perception in noise)] and [(advantages) or [(disadvantages)]. First, the authors read the titles and abstracts of the articles and selected those that were interesting while being as inclusive as possible. Following the first phase, they independently assessed the full text of all potentially relevant studies for inclusion in this review. Any disagreement was resolved through discussion with a third author (biostatistician, N.S.). Then, using a standardized data collection form, the following information was extracted from the included studies: first author, journal, publication year, title, database, study aim, type of study and sample size, and results. 

The inclusion criteria were primary research studies (including descriptive and observational studies, randomized trials, and basic science articles) published after October 2020 on the advantages and disadvantages in adult patients with BS on speech perception in noise. This choice to select recent publications was due to the impact of updated programming software growth in this field and, consequently, to the greater data relevance of the recent publications. We excluded those that did not fit the inclusion criteria or that directly addressed the topic under investigation; in particular, we excluded all articles that referred to bilateral cochlear implantation and other special cases of patients with syndromic diseases.

## 3. Results

**Figure 1:**
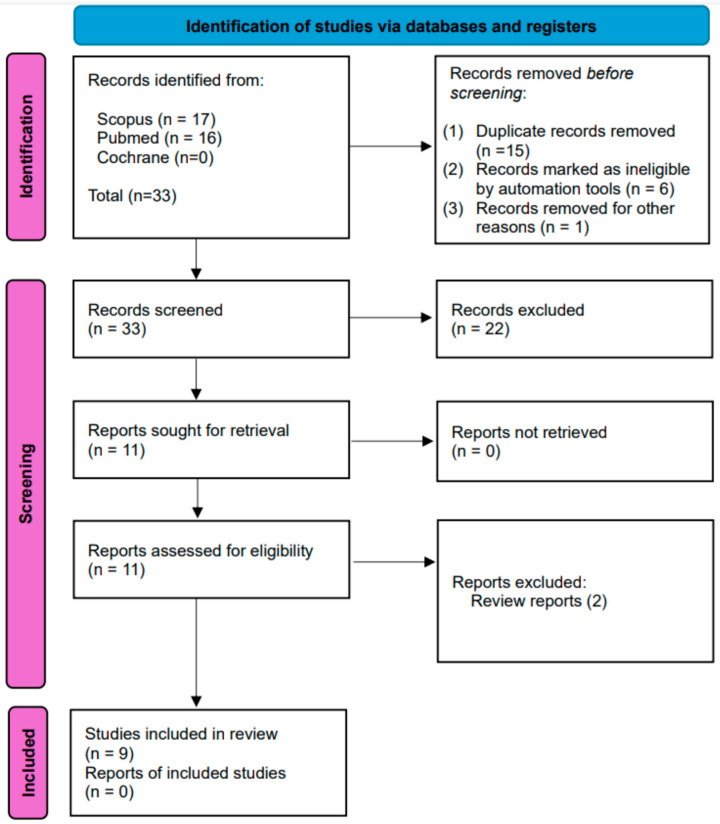
PRISMA flow diagram 2020. The flowchart displays article search and selection. PRISMA flow diagram 2020. The flowchart displays article search and selection according to MJ, McKenzie et al. [10].

Overall, our search generated 33 articles. We removed 15 articles as duplicate records, 6 articles that did not meet the inclusion criteria or deal directly with the issue investigated and 1 article for other reasons (Deutsch language). As a result, our systematic search provided 9 articles. Details of the search performed are shown in the flowchart in Figure 1.

In Table 2, the selected articles are summarized.

### The Quality Assessment

Two reviewers (R1 and R2) have independently conducted the quality assessment of the articles included in our study. Both reviewers were researchers with expertise in reviewing methods and critical appraisal, rousing the Effective Public Health Practice Project Quality Assessment Tool (EPHPP) [15]. The two researchers expressed an opinion on each individual article. Subsequently, the opinions were assessed by a third reviewer represented by a biostatistician, who expressed the final assessment. EPHPP tool was developed for systematic reviews of public health topics and can be used in different study designs and is characterized by six components: “selection bias”, “study design”, “confounders”, “blinding”, “data collection methods”, and “withdrawals and drop-outs”. The partial and global score assigned for the assessment of quality level were Weak, moderate, or strong [available at: https://www.ephpp.ca/quality-assessment-tool.html; last access: 19 July 2024]. In Table 3, the quality of all included studies was reported.

## 4. Discussion

The real advantages and disadvantages in adult patients with BS, compared with bilateral CIs, on speech perception in a silent environment or with background noise are still controversial. Based on the literature revision, the BS is considered a solution in individuals with severe, profound sensorineural hearing loss in cases where they do not meet the criteria for bilateral CIs [11]. Despite there being common evidence that BS improves verbal abilities as compared to unilateral CI, few reports quantify speech perception outcome of BS. In particular, the greatest gains occurred in the first 3 months and slightly continued up to 12 months in adult patients [11,12,16].

Thus, in patients who the HA failed to provide adequate speech recognition after appropriate HA fitting, CI candidacy for bilateral CIs is recommended [12]. Other findings indicate that residual hearing in the ear contralateral to the CI provides even greater benefit in consonant perception when visual cues are also available (trimodal speech perception), thus emphasizing the importance of preserving residual low-frequency acoustic hearing in CI candidates through alternative surgical approaches and/or electrode designs that increase the likelihood of hearing preservation [17]. In addition, a recent systematic review focusing on the analysis of the benefits of hearing preservation in cochlear implants confirmed the importance and benefits of residual hearing post-surgery in subjects with BS [1]. However, even the presence of residual hearing in the better ear is a critical point for the candidacy to BS as compared to unilateral stimulation. Indeed, based on these broader CI criteria, such as the better hearing threshold for the indication to cochlear implantation, the number of BS users is expected to rise globally [7].

Ideally, two-ear listening should improve speech recognition, sound quality, spatial hearing, and quality of life while reducing the listening effort and fatigue that accompanies continuous listening [6].

Individuals who use BS experience discrepancies in the timing of stimulation due to varying processing delays between the two devices. This disparity in device timing results in a temporal misalignment in the stimulation of the auditory nerve. Correcting this misalignment by addressing the differences in device delays can significantly improve the accuracy of sound source localization. Such enhancements are attributed to binaural auditory fusion, which involves central mechanisms that integrate signals from both ears, thereby improving speech perception in noisy environments [4,5]. On the contrary, in a recent review article addressing the interaural mismatches between electric and acoustic stimulation, Zirn et al. [18] sustained that the technical compensation of the interaural sustained latency offset was useful only on sound localization ability, while data did not support the beneficial effects on the speech understanding in noise in bimodal listeners.

Several pieces of evidence suggest that the benefits of BS in verbal perception are undermined by amplitude-modulated noise, which increases difficulties in comprehension, or how “temporal fine structure” (TFS), which refers to the rapid fluctuations in the phase of a sound wave that occurs over time, which are critical for processing complex sounds, such as speech and music recognition, which influences in temporal modulation is facilitated by HAs. TFS cues provide important information for sound localization, pitch perception, and understanding speech in noisy environments [13,19]. The extensive use of dichotic listening tests could be useful in fitting the device to maximize results. In addition, Gifford et al. showed in a multicenter study an improvement of speech perception in noise in EAS and BS than with a unilateral CI. Unfortunately, the results in the current literature remain controversial about the better performance of EAS and BS compared to CI unilateral. Further study indicates beneficial effects of BS/EAS in the case of ski-slope audiogram for the preservation of the lower frequencies [6,8,9].

BS technological improvement has been introduced, including directional microphones that are able to ensure adequate reduction of ambient noise [20] and dedicated CI programming software that can minimize the processing temporal delay between a CI and contralateral HA, thereby improving sound localization [4,5,21]. Additionally, as indicated above, the R-SPACE™ sound system can enhance the assessment of speech perception in noise [22,23].

Increasing data indicate that a marker for binaural hearing outcomes is the higher results of speech audiometry tests compared to the use of CI alone [11,12,16,19]. Accordingly, to the aim of this systematic review, verbal perception in a noisy environment can be usefully evaluated with musical sound perception tests. It is well known that BS obtains beneficial effects on music listening, so these tests can provide additional information to speech audiometry tests, and the results of the music perception tests appear to be related to hearing-related quality of life [23].

Furthermore, during the last years, several studies [24,25,26] have demonstrated significant CI and bimodal benefits in adults with asymmetric hearing loss (AHL), i.e., moderate-to-profound hearing loss in one ear and better hearing in the other ear. Individuals who have AHL experience reduced sound quality and increased effort during real-life listening conditions that include noise, reverberation, multiple speakers, and distance [27] due to partial or complete loss of binaural hearing advantages, such as binaural squelch, head shadow effects, and binaural loudness summation. The standard treatment options for asymmetric hearing with amplification to the poor ear, a contralateral routing of the signal (CROS/BiCROS) hearing aid (HA), or a bone-anchored hearing aid (BAHA) device are rarely successful solutions because these strategies do not restore the perception of binaural cues. On the contrary, benefits have been reported in AHL subjects with a CI system on one side and a hearing aid on the other while hearing in quiet conditions, especially when listening to soft sounds and voices, but mostly while hearing in noise. In addition, gains in sound localization, mainly in cases with a short hearing deprivation duration and with post-lingual hearing loss, have been demonstrated, as well as benefits to quality of life [28]. Particularly, subjects with low-frequency hearing thresholds less than 60 dB HL in the non-implanted ear showed greater bimodal benefit in noise than participants with low-frequency thresholds greater than 60 dB [29,30]. Currently, FDA guidelines restrict cochlear implantation in AHL to those with profound hearing loss and very poor, aided word recognition in the PE, thus excluding moderate-to-profound hearing loss and duration of profound hearing loss < 10 years. Therefore, the onset and duration of hearing loss are factors to consider in this population. Indeed, despite the positive results, the outcome variability among CI recipients has been well documented [31,32,33,34]. The contributory factors have been identified in speech perception scores before implantation, the degree of hearing loss in the non-implanted ear, and the length of hearing deprivation in the implanted ear. But recently, it has been reported that, in adults with post-lingual asymmetric hearing loss, a long duration of sound deprivation in the ear to be implanted has little influence on speech recognition outcomes after receiving a cochlear implant. Thus, the variability in the outcome between patients with asymmetric hearing loss could be only partially explained by the degree of hearing loss or by the length of hearing deprivation but also by the integration process that is highly listener-specific and based on individual features. Recently, Firszt et al. [34] conducted a multicenter, FDA-approved clinical study to better define CI candidacy guidelines for individuals with AHL, adopting study inclusion criteria for the PE that are less restrictive than current FDA candidacy criteria. This, as well as other studies, indicate that clinicians should consider a CI for individuals with AHL, if the PE has a PTA (0.5, 1, 2 kHz) > 70 dB HL and a CNC word score ≤ 40%. LOD > 10 years should not be an exclusion. These findings show the necessity to expand the current FDA criteria for AHL. Mancini et al. [14] have confirmed the benefit of bimodal stimulation in terms of speech perception scores compared to CI alone, even in elderly patients with asymmetric hearing loss when evaluating the outcomes of bimodal listening in the elderly through adaptive tests (STARR and Matrix). The role of aging is known to negatively affect auditory performance and compromise the central decoding process [35]. In this study, the duration of deafness does not affect the results of bimodal stimulation in patients with a minimum bimodal hearing of 1 year and an average of 4.9 years. The duration of CI/HA experience probably determines the benefit of bimodal stimulation, providing the time necessary to adapt to the new listening mode.

Even though BS has been extensively proposed in adult patients with asymmetric hearing loss for its beneficial effect on localization ability and mainly on speech in noise perception, other relevant aspects can influence the outcomes of BS. A large body of evidence supports the advantages of BS, especially if compared with unilateral CI in adults, mainly on pleasant music listening [36], overall quality of life [14], and the domain of social activities [37]. Nowadays, an increasing number of studies link hearing loss to cognitive decline, especially in the aged population [38], while multiple studies have demonstrated long-term improvement of cognitive functioning in several domains: learning and memory, language, perceptual-motor function, executive function, and attention in older adults after hearing rehabilitation with both HAs and CIs [39,40]. Although a lot of literature is available on the positive effects of CI on cognition, a critical report by Hua et al. [41] indicates the possibility that bimodal listening might require increased cognitive skills as compared to unimodal CI listening because of the difficulties of integration of two different signals generated by HAs and CIs, mainly in noisy environment. Further studies will be useful in understanding this relevant topic for the use of bimodal listening. The choice of BS is also largely discussed for children’s hearing rehabilitation. Not that long ago, the unilateral CI was indicated in children because they faced the risk of detrimental effects in their language development as a result of bilateral severe-to-profound hearing loss, without the beneficial effects of HAs and speech therapy training on language development [42]. However, simultaneous bilateral CI is currently recommended as cost-effective in children with severe-to-profound sensorineural deafness based on the evidence of the advantages on speech perception in quiet and noise and sound localization [43]. Bimodal listening can still be provided in children with asymmetric hearing or residual hearing, avoiding the harmful effect of unilateral stimulation on the maturational changes leading to the abnormal cortical preference for the stimulated ear, which can persist if a second CI is delayed [44,45]. However, in addition to benefits in various listening situations in everyday life and music perception, a period of bimodal hearing can facilitate the acquisition of language abilities in children based on the possibility of having access to acoustic speech signals as low-frequency signals that children with normal hearing use during the earliest stages of language acquisition [46]. Interestingly, Gifford [47] confirmed that bilateral CIs yield superior outcomes for children with bilateral severe-to-profound hearing loss; however, an early period of bimodal stimulation can be useful for speech perception and language development only for children with better-ear PTA ≤ 73 dB HL [47]. Despite the myriad of data in favor of the early bilateral cochlear implant, a recent review comparing outcomes in children who had a CI or were using HAs raised a number of doubts on the interpretation of the overall outcomes underlying the opportunity to fill the gap of knowledge on the beneficial effects of CIs and HAs in various auditory scenarios [48]. Although worldwide guidelines recommend early simultaneous bilateral CIs in children, the BS in some situations should be taken into account, while it remains a great opportunity in adults for the amount of beneficial effects in in real-world listening situations.

Although the evidence of the beneficial effects of bimodal listening in adults is clearer than in children, the outcome with respect to bilateral CI is still controversial. Some limitations on the benefits of BS compared to bilateral CIs have been reported. Firstly, there is significant variability in patient outcomes and study methodologies, making difficult general conclusions [16,49,50]. The degree of residual hearing plays a crucial role, as those with more residual hearing tend to benefit more [1]. Technological differences between ICs and HAs in BS complicate the comparisons. Despite new technologies, such as updated programming software and improved compensation for temporal delay between devices, showing promise in addressing these issues, their real-world application remains underexplored. In addition, the lack of suitable patient selection criteria and standardized audiological assessment represent further critical concerns. Lastly, most studies focus on short-term outcomes; thus, long-term research to better understand sustained benefits and overall patient satisfaction is desirable.

Below, we summarize in detail the main advantages and disadvantages of our study.

The main advantage of BS is speech perception, which is qualitatively better compared to the use of the unilateral CI alone in both quiet and noisy environments, mainly increasing for subjects who have retained more residual hearing. The most relevant prognostic factor is residual hearing in the implanted ear [1], especially in the low frequencies. Additionally, residual hearing in the contralateral ear undergoing HA application greatly favors the recognition of consonants [8]. Interestingly, this effect can be amplified with the use of visual cues, resulting in a multidimensional perception referred to as trimodal stimulation [17]. Further benefits have emerged by using new technologies such as directional microphones and programming software that reduce ambient noise and minimize processing delays between ICs and HAs [20], thus improving spatial streaming capabilities [51]. Assessment of hearing performance and quality of life is made more comprehensive by using several “real-life” tools such as R-SPACE™, a system that allows audiometric tests to be conducted under specific conditions by simulating “real-life” noisy environments and music perception tests providing valuable information [22,23,52].

Nevertheless, one drawback that must be considered is modulated background noise that can interfere with the BS stimulation, making speech comprehension more difficult [13]. Thus, the technical complexity of bimodal fitting is challenging [53]. Optimizing the performance, balance, and integration of the devices requires advanced technology, precise programming, and professional expertise. Thus, the candidacy for bilateral CIs versus BS stimulation can be multifaceted based on reliable evaluation tools.

Nowadays, there are no clear guidelines in the literature with respect to the selection criteria for choosing patients who are candidates for a BS stimulation system, which is a prerequisite for more advantageous and reliable results and outcomes.

Finally, we included in the revision of literature the last 5 years, considering recent progress on the inter-device coupling systems and new insight on the reduction of time gap between devices improved quality of life in BS users that were not addressed in previous review studies including articles published over the past decade. However, all reports [1,54] confirm the benefit of BS over unilateral stimulation in improving speech noise perception. Therefore, every effort should be made to improve technical approaches for the binaural balance of the two devices, and it is recommended to encourage CI recipients to continue using contralateral HA after CI. Schaefer S et al. [1] focus on the importance and benefits of preserving residual hearing in CI patients and how these benefits are associated with better perception of speech in noise and better perception of music. Interpretation of the results, however, was hampered by significant heterogeneity in various parameters. Karimi-Boroujeni M et al. [54] deals with the effects of hearing disorders on prosody perception in children and adults and evaluates the performance of devices in restoring prosodic perception. On the contrary, we focused on the advantages and disadvantages of BS in adult patients on speech perception in noisy environments, and data suggest that the coupling systems between CI/HA devices is crucial in reducing the temporal gap in the interpretation of acoustic information between the two sides.

## 5. Conclusions

BS offers significant benefits in speech perception in quiet and noisy environments in adult patients, especially for those with residual hearing. Preservation of residual hearing and the use of advanced technologies, such as programming software that can reduce environmental noise and systems aimed at compensating the temporal gap between the two devices (ICs and HAs), further improve outcomes [4,5,20]. However, modulated noise may limit these benefits. Studies indicate that electro-acoustic stimulation (EAS) outperforms BS hearing in noise [6,8,9,52]. Assessment tools such as R-SPACE™ and music tests provide additional information on hearing performance and quality of life, confirming the importance of binaural hearing [22,23]. Nowadays, there are no definitive guidelines in the literature with respect to the selection criteria for choosing patients who are candidates for a BS system, which is a prerequisite to achieving beneficial and reliable outcomes in terms of speech perception in noise. Taken together, the literature confirms the recommendation to encourage CI recipients to continue contralateral HA use after CI, and provides evidence of the beneficial effects of the coupling of CI/HA devices, focusing on the reduction of the temporal gap in the interpretation of acoustic information between the two sides in optimizing speech perception in noise which remains a challenging issue.

## Figures and Tables

**Table 1:** Database, search strings, and number of articles found.

Database	Search Strings	Number of Results
PubMed Central	(acoustic bimodal stimulation on speech perception in noise) AND ((“1 October 2020”[Date—Publication]: “17 July 2024”[Date—Publication]))	16
Cochrane	acoustic bimodal stimulation on speech perception in noise in Title Abstract Keyword—with Cochrane Library publication date Between Oct 2020 and Jul 2024 (Word variations have been searched)	0
Scopus	TITLE-ABS-KEY (acoustic AND bimodal AND stimulation AND on AND speech AND perception AND in AND noise) AND PUBYEAR > 2019 AND PUBYEAR < 2025	17

**Table 2:** Description of included papers.

First Author, Title (IT/EN), Journal, Year	Database	Topic Aim of the Study	Type of Study, Sample Size, Tools	Results
Fan Y, Objective measure of binaural processing: Acoustic change complex in response to interaural phase differences. Hear Res. 2024 [4]	Pubmed	To investigate the feasibility of using an objective measure of interaural phase difference (IPD) sensitivity via the acoustic change complex (ACC) to assess candidacy for cochlear implantation with preserved low-frequency acoustic hearing, focusing on its correlation with behavioral interaural time difference (ITD) sensitivity and its potential clinical application.	Type of study: Experimental study. Sample size: Ten adult listeners with normal hearing. Tools: Behavioral and objective measures of binaural cue sensitivity using the acoustic change complex (ACC) with imposed IPD at 250 Hz and 1000 Hz.	(1) ACC amplitude increases with IPD; (2) Significant correlation between ACC-based IPD sensitivity at 250 Hz and behavioral ITD sensitivity; (3) Higher sensitivity to IPDs observed at 250 Hz compared to 1000 Hz, suggesting potential clinical utility for identifying EAS candidacy.
Gifford RH, Cochlear implant spectral bandwidth for optimizing electric and acoustic stimulation (EAS). Hear Res. 2022 [6]	Pubmed	The study explores the impact of different crossover frequencies in electric and acoustic stimulation (EAS) on speech recognition and listening difficulty in cochlear implant users with acoustic hearing preservation. It compares CI alone, BS (CI+HA), and best-aided EAS (CIHA+HA) listening conditions.	Type of study: Exploratory study. Sample size: 15 adult cochlear implant recipients with acoustic hearing preservation. Tools: Speech recognition test in semi-diffuse noise, subjective listening difficulty estimates.	The study found no significant effect of low-frequency cochlear implant (CI) cutoff on speech recognition or subjective listening difficulty across three listening conditions: CI alone, BS (CI+HA), and best-aided EAS (CIHA+HA). Both BS and best-aided EAS conditions showed significant benefits over CI alone, with additional benefits observed in best-aided EAS compared to BS hearing. Future research is needed to explore the efficacy of spiral ganglion (SG)-place-based fitting strategies for optimizing outcomes in experienced and newly activated EAS users.
Mertens G, Contralateral hearing aid use in adult cochlear implant recipients: retrospective analysis of auditory outcomes. Int J Audiol. 2024 [7]	Pubmed	To retrospectively investigate the frequency of usage of BS among cochlear implant (CI) users, as well as its clinical benefit relative to unilateral use.	Type of study: Retrospective study. Sample size: 103 adults Tools: Clinical Minimal Outcome Measurements test battery.	The preoperative contralateral residual hearing in the BS group was significantly better than that of the CI-only group. In both groups, speech perception in quiet and in noise improved after CI, with no significant difference between postoperative unimodal conditions. For the BS group, an additional significant improvement was found for the BS condition compared to the unimodal.
Yoon YS, Effects of the Configuration of Hearing Loss on Consonant Perception between Simulated Bimodal and Electric Acoustic Stimulation Hearing. J Am Acad Audiol. 2021 [8]	Pubmed	Investigating the impact of different configurations of hearing loss on speech perception benefits in BS and electric acoustic stimulation (EAS) hearing. To determine how consonant recognition is influenced by various patterns of hearing loss in simulated BS and EAS conditions using acoustic stimulation.	Type of study: Experimental study. Sample size: 20 adult subjects (10 per group). Tools: Acoustic simulation for consonant recognition, band-pass filtering for simulated hearing loss configurations, eight-channel noise vocoder for electric stimulation mimicking spectral mismatch.	Significant BS and EAS benefits occurred regardless of the configurations of hearing loss and hearing technology (BS vs. EAS). Place information was better transmitted in EAS hearing than in BS hearing.
Yoon YS, Interactions Between Slopes of Residual Hearing and Frequency Maps in Simulated Bimodal and Electric Acoustic Stimulation Hearing. J Speech Lang Hear Res. 2024 [9]	Pubmed	Topic: Investigating the influence of residual hearing slopes and cochlear implant frequency map settings on BS and electric acoustic stimulation (EAS) benefits in speech perception. Aim: To determine how different configurations of residual hearing and frequency map settings impact the benefits of BS and EAS stimulation in speech perception tasks.	Type of study: Experimental study Sample size: Adults with normal hearing. Tools: Sentence perception tests (unilateral and bilateral), acoustic stimulation with low-pass filters for hearing loss slopes, eight-channel sinewave vocoder for electric stimulation with different frequency map settings.	The largest BS/EAS benefit occurred with the shallow slope, and the smallest occurred with the steep slope. The effects of the slopes on BS/EAS benefit were greatest with the meet or gap map and the least with the overlap map. EAS benefit was greater than BS benefit at higher signal-to-noise ratios regardless of frequency map.
Dourado RPB, Benefits of Bimodal Stimulation to Speech Perception in Noise and Silence. Int Arch Otorhinolaryngol. 2023 [11]	Pubmed	To present whether bimodality still offers hearing benefits to the population who uses acoustic stimulation associated with electrical stimulation.	Type of study: Observational study. Sample size: 13 participants Tools: Hearing in Noise Test (HINT), Visual Analog Scale (VAS), four-tone means, Speech, Spatial, and Hearing Qualities questionnaire.	Individuals with an average hearing level between 50 and 70 dB showed better sentence recognition in both silence and noise.
Kelsall D, Longitudinal outcomes of cochlear implantation and bimodal hearing in a large group of adults: A multicenter clinical study. Am J Otolaryngol. 2021 [12]	Pubmed	To evaluate speech understanding outcomes in post-linguistically deafened adults with poor hearing performance despite well-fit hearing aids, comparing unilateral and bilateral/BS listening conditions preimplant and up to 12 months post-implant.	Type of study: Multicenter, prospective, repeated-measures, within-subject controlled study. Sample size: 100 post-linguistically deafened adults with bilateral moderate-to-profound sensorineural hearing loss. Tools: Speech recognition tests: monosyllabic consonant-nucleus-consonant (CNC) words in quiet; AzBio sentences in coincident noise at +5 dB and +10 dB SNR in implant ear and BS conditions.	Significant improvements in monosyllabic word scores in the implant ear only: 84% at 3 months, 93% at 6 months, and 97% at 12 months post-implant. Mean gain of 51% points for monosyllabic words and 32% points for sentences in noise at 12 months (*p* < 0.001). BS condition: 87% demonstrated improved monosyllabic word scores at 6 months, with a mean gain of 40% points (*p* < 0.001). Significant improvements in sentences in noise at 6- and 12-months post-implant in the BS condition (*p* < 0.001).
Stronks HC, The Temporal Fine Structure of Background Noise Determines the Benefit of Bimodal Hearing for Recognizing Speech. J Assoc Res Otolaryngol. 2020 [13]	Scopus	To investigate the role of temporal fine structure (TFS) and envelope cues in speech recognition in noise for cochlear implant (CI) users and BS listeners.	Type of study: Experimental study. Sample size: Not specified in the text. Tools: Speech recognition tests in steady-state (SS) noise, babble noise, and amplitude-modulated steady-state (AMSS) noise.	Babble noise was more detrimental to speech recognition than AMSS noise in CI-only conditions, contradicting initial hypotheses about TFS availability. BS benefit was observsed across noise types, indicating TFS dependence rather than envelope cues alone.
Mancini P, Bimodal cochlear implantation in elderly patients. Int J Audiol. 2021 [14]	Pubmed	Bimodal stimulation for asymmetric hearing loss in elderly adults, focusing on benefits in speech perception integration, particularly noise environments.	Type of study: retrospective clinical study. Sample size: 17 bimodal cochlear implant users. Tools: Speech audiometry in quiet and noise conditions using STARR and Matrix tests.	Bimodal Pure-tone threshold audiometry and speech perception in booth quiet and noise conditions significantly outperformed CI or HA alone. Age had a significant effect on bimodal STARR results, and bimodal STARR scores improved significantly compared to the better ear condition.

**Table 3:** Quality assessment: EPHPP scores.

Author, Year, [Ref. num]	EPHPP Scores												
	SB		D		C		B		DC		DO		Overall
	R1	R2	R1	R2	R1	R2	R1	R2	R1	R2	R1	R2	
Fan Y, 2024 [4]	W	W	W	W	W	M	W	W	S	S	S	S	W
Gifford RH, 2022 [6]	W	W	W	W	W	W	W	W	M	M	S	S	W
Mertens G, 2024 [7]	S	S	W	W	S	S	n/a	n/a	S	S	S	S	S
Yoon YS, 2021 [8]	M	S	W	W	W	W	W	W	M	W	S	S	W
Yoon YS, 2024 [9]	W	W	W	W	W	W	W	W	W	M	S	S	W
Dourado RPB, 2023 [11]	W	W	W	W	W	W	n/a	n/a	S	S	S	S	W
Kelsall D, 2021 [12]	S	S	M	M	S	S	W	W	S	S	S	S	S
Stronks HC, 2020 [13]	W	W	M	M	W	W	S	S	S	S	W	W	W
Mancini P, 2021 [14]	W	W	W	W	W	W	n/a	n/a	S	S	S	S	W

SB = Selection Bias, D = Study Design, C = Confounders, B = Blinding, DC = Data Collection Method, DO = Withdrawals and Dropouts, W = Weak, M = Moderate, S = Strong, n/a = Not applicable.

## Data Availability

Data sharing is not applicable.
